# A special issue preface: diamond for quantum applications

**DOI:** 10.1098/rsta.2022.0323

**Published:** 2024-01-22

**Authors:** Shannon S. Nicley, Gavin W. Morley, Ken Haenen

**Affiliations:** ^1^ Department of Electrical and Computer Engineering, Michigan State University, East Lansing, MI, USA; ^2^ Department of Physics, University of Warwick, Coventry, UK; ^3^ Institute for Materials Research (IMO), Hasselt University & IMEC vzw, Diepenbeek, Belgium

**Keywords:** diamonds, quantum systems, colour centres

## Abstract

This special issue discusses current progress in the utilization of defect centres in diamond as spin–photon interfaces for quantum applications. This issue is based on the discussions of the Theo Murphy meeting ‘Diamond for quantum applications' which covered the recent progress of diamond growth and engineering for the creation and optimization of colour centres, toward the integration of diamond-based qubits in quantum systems.

This article is part of the Theo Murphy meeting issue 'Diamond for quantum applications'.

## 1. Introduction

Quantum theory has revolutionized our understanding of the interactions of light and matter at the nanoscale. The discovery of these effects led to a first quantum revolution, which brought advances such as transistors and LEDs, changing our world immeasurably. Our ability to control quantum mechanical phenomena in customized systems is now starting to be realized in a second quantum revolution, where the technological capabilities of quantum mechanics are fully implemented in devices with fundamentally superior performance in computing, sensing, imaging, simulation and communication. Diamond, as a solid-state host material for optically active defects, has the potential to accelerate this second revolution, with far-reaching social implications. These include safer encryption of data and an acceleration of biological research due to both advances in quantum sensing and quantum computing simulations of biological systems like protein folding. More efficient calculation of problems like minimizing travel routes and quantum-aided search algorithms could also have an enormous impact in terms of energy conservation. This special issue is based on a Royal Society Theo Murphy Meeting, which took place 10–11 October 2022, with the goal of accelerating the development of diamond as a quantum material by promoting the interaction of the key research fields in this area at a critical time in its development.

Ongoing rapid research progress using optically active colour centres such as the nitrogen vacancy (NV) centre has shown diamond's significant potential for quantum applications. The recent demonstration of electronic readout of an electronic spin state of a single NV centre [1] shows the potential for device fabrication for various quantum applications. These include magnetic field sensing, such as the recent achievement of high sensitivity high-frequency sensing using dynamical decoupling [2], and sensitive magnetometry with a fibre-coupled sensor [3]. The demonstration of repeated quantum error correction also proves NV centres are suitable as the basis for scalable quantum computation [4].

Diamond, as a solid-state qubit host, offers major advantages over more technologically mature platforms, like superconducting qubits and ion traps. These include less restrictive temperature requirements and scalable two-dimensional arrays of qubits which can be extended to the approximately 10^5^ qubits estimated needed for useful quantum computing. The lively discussion at this meeting and summary of the outstanding issues in achieving ambitious quantum devices (such as 100 000 qubit diamond-based quantum computers) was therefore extremely timely. One issue is the low collection efficiency of indistinguishable photons from the NV centre. Device solutions such as optically coupled open microcavities were presented, as well as nanofabrication techniques for accurately positioning NV centres in such structures, such as multiphoton laser writing [5]. Furthermore, the electronic structure of new optical defects, which show promise for improving indistinguishable photon emission performance [6], were also discussed in great depth. Experimental investigations of these new centres, such as the tin vacancy (SnV) centre [7,8] and nickel vacancy centre [9], also illuminated a common need for charge state control of these centres and demonstrated that the field of optically active colour centres in diamond is still rapidly expanding, as evidenced in the context of this Special Issue.

## 2. Contents of the theme issue

The NV^−^ centre in diamond has been the most widely studied of the crystalline defects in diamond for quantum applications. This special issue also reflects this focus, with half of the papers looking at the optimization, use and deeper understanding of the NV defect. Two papers looked specifically at the properties of NV centres in diamond related to the nitrogen concentration of diamond grown by chemical vapour deposition (CVD) toward the optimization of the NV performance. Luo *et al.* [10] study diamond absorption and birefringence, with the aim of minimizing both while maximizing the NV concentration. Reducing these optical losses is essential for applications like diamond lasing, optical threshold magnetometry, diamond cavity applications, high-power applications and polarization-dependant applications of NV diamonds. Teraji *et al*. [11] summarize the state of the art for the growth of nitrogen containing diamond material, as well as techniques for the formation of the NV^−^ centre, with the aim of maximizing the T_2_ coherence times. They describe a method for highly controlled nitrogen incorporation for the optimization of the magnetic field sensitivity of devices based on the grown NV diamond material.

The use of highly sensitive NV diamond for magnetometry in real world environments is also an area of current research interest. Hatano *et al.* [12] describe one such application, where the diamond quantum sensors based on NV centres are used as an electric vehicle battery monitor. They characterized the performance and noise in a real car and compared the prototype diamond sensors favourably or on par with conventional sensors such as Hall sensors and shunt resistors.

A greater understanding of the physics underlying the creation of NV defects in diamond is also needed, to enable the full optimization of bulk NV diamond material as well as the properties of single centres formed with high spatial precision. The creation of NVs necessitates the creation of vacancies in the diamond, in the form of Frenkel defects (vacancy-interstitial pairs), both components of which can interact with the formed NV centres, affecting their properties. Kirkpatrick *et al*. [13] use density functional theory (DFT) methods to investigate the strain and electronic interactions between the NV^−^ centre and the self-interstitial in diamond. They propose that the formation of a hybridization between the NV's electronic structure manifold and the π-bonds of the interstitial may form non-radiative channels allowing the excited electron to return to the ground state, which explains the fluctuating fluorescence signal observed during annealing by femto-second laser processing.

Two of the other papers also used DFT methods, however moving away from the issue's initial focus on NV material, they have investigated the properties of crystal defects beyond the NV centre in diamond. Thiering & Gali [14] look specifically at the spin-orbit and Jahn–Teller interactions of the substitutional nickel defect in diamond. Through this framework, the study associates a previously reported photoluminescence centre at 2.51 eV and an optically detected magnetic resonance centre at 2.56 eV to the negatively charged substitutional nickel defect and the emission from the bound exciton excited state of the neutral defect, respectively. These assignments allow for deeper experimental detection and investigation of these centres. Morris *et al.* [15] study crystal defects in diamond containing radioactive rare isotopes such as ^229^Pa, as well as defects containing stable analogues. These defects may be used in conjunction with quantum control methods to perform experimental tests of fundamental symmetry violations.

The experimental investigation of novel defects in diamond beyond the NV centre was a central focus of the Theo Murphy meeting and is represented in this special issue in two papers. Sedov *et al.* [16] produced tin-vacancy colour centres in diamond (SnV) by CVD using SnO_2_ particles as the source of the tin impurity. They studied the optical properties of the grown material by photoluminescence, and attributed changes in the shape of and position of the zero-phonon line of the SnV to the effect of strain in the grown microcrystals. Boldyrev *et al*. [17] use spectroscopic methods to study the isotopic shift in SiV^0^ centres. Their findings included that for potential quantum applications of the SiV^0^, using isotopically pure carbon is more important than pure silicon for achieving a narrow spectral line.

## Data Availability

This article has no additional data.
